# Recognition of the Presence of Bone Fractures Through Physicochemical Changes in Diagenetic Bone

**DOI:** 10.1177/00037028231213889

**Published:** 2023-11-13

**Authors:** Caley Mein, Jennifer R. Jones, Catherine Tennick, Anna Williams

**Affiliations:** 1 Research Centre for Field Archaeology and Forensic Taphonomy, School of Law and Policing, 6723 University of Central Lancashire, Preston, UK

**Keywords:** Trauma, bone fractures, bone diagenesis, physicochemical composition, attenuated total reflection Fourier transform infrared spectroscopy, ATR FT-IR, scanning electron microscopy energy dispersive spectroscopy, SEM-EDS

## Abstract

Much research has focused on attempting to understand the drivers of bone diagenesis. However, this sensitive process is easily influenced by various factors, particularly the condition of the remains (i.e., whether they have been subjected to trauma). Previous research demonstrates that trauma can influence soft tissue decomposition, yet to date, no studies have looked at how bone fractures could affect bone diagenesis. To address this gap, two short timescale studies were conducted to investigate the influence of bone fractures on the physicochemical composition of disarticulated, partially fleshed animal remains. Disarticulated porcine bones were either fractured using blunt force or sharp force whilst fresh (producing perimortem damage), at 60 days producing postmortem damage (postmortem interval (PMI)), or left intact and left outside for up to 180 days post-fracture/240 days PMI. Retrieved bone sections were then analyzed for physicochemical differences using non-destructive methods, i.e., scanning electron microscopy energy dispersive spectroscopy and Fourier transform infrared spectroscopy with attenuated total reflectance. It was hypothesized that differences would be found in the physicochemical composition between the bones with fractures and those without after undergoing diagenetic change. The bone fractures significantly affected the elemental composition of bone over time, but structural composition initially remained stable. It was also possible to distinguish between perimortem and postmortem fractures using these two analytical techniques due to physicochemical differences. This research shows bone fractures can significantly alter the physicochemical composition of the bone during the postmortem period and have the potential to facilitate more accurate PMI estimations in forensic contexts.

## Graphical abstract

**Graphical abstract:**
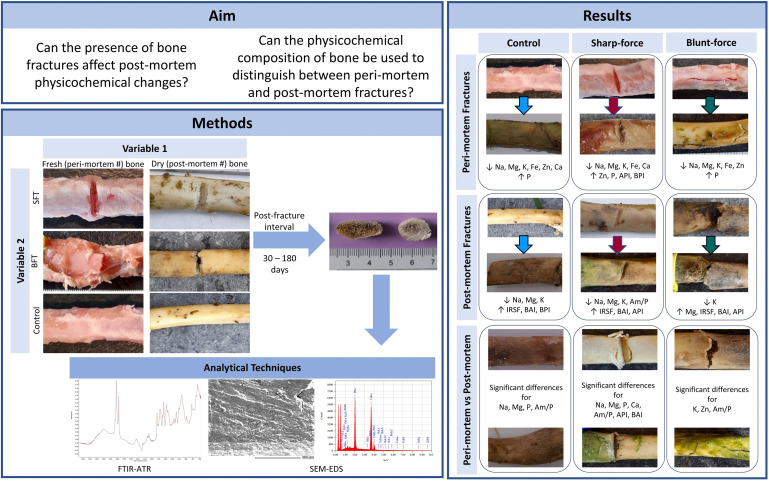
This is a visual representation of the abstract.

## Introduction

Physical trauma is increasingly seen in police investigations; there were 1.5 million violent incidents against people aged 16 and over for the year ending March 2022 in the UK.^1^ Although incident numbers decreased by 10% in 2021 compared to 2020, most likely as a result of the pandemic and lockdown restrictions, these numbers have since risen to pre-pandemic levels.^1^ Of these 1.5 million incidents, almost 50 000 involved knives or other sharp implements.^1^ There were 710 homicides recorded by the police, 40% of which were classed as sharp-force trauma (SFT).^1^ An abundance of literature is available on weapon identification from trauma marks in bone,^2–5^ on clothing,^6,7^ and identification from particles left behind by the weapon,^8,9^ but very little is available on how bone damage from trauma could influence the postmortem changes that bone can undergo and how these changes could affect potential postmortem interval (PMI) estimation techniques.

### Background

Bone is a composite material comprised of both organic and inorganic fractions.^10,11^ The organic component is approximately 90% collagen, which provides bone with its flexibility, while the inorganic component, a hydroxyapatite, gives bone its rigidity.^12,13^ Bone diagenesis refers to the physical and chemical changes that occur to skeletal tissue after death. These changes can lead to fossilization or destruction of the bone.^14–20^ The majority of the literature exploring diagenesis focuses on archaeological bone and the fossilization process,^16,19–25^ and the diagenesis of bone for isotope analysis.^26–29^ In recent years, the focus has shifted to exploring shorter timescales and the possibility of developing more accurate methods of PMI estimation when dealing with skeletonized remains,^30–34^ but so far due to the complex nature of decomposition, accurate PMI estimation methods remain elusive. Three main pathways have been identified as the drivers of bone diagenesis; microbial attack through bioerosion, loss of the organic component through hydrolysis, and loss of the inorganic component through dissolution.^18,21^ The condition of the remains (presence of infection, trauma, blood loss, and dismemberment) can also affect the extent of diagenetic change,^35^ so it is important that different conditions are studied. This research hypothesized that bone fractures could influence diagenetic change as the compromised skeletal material could allow easier access to microbes, water movement, etc., and these could impact the accuracy of any PMI estimation techniques.

During the post-depositional period, bone can undergo changes within the crystal lattice resulting in alterations to its physicochemical composition. It has been hypothesized that understanding the physicochemical composition of bones could potentially aid PMI estimation and help to determine whether remains have been moved from the primary deposition site.^34,36^ Research in this area is crucial as more accurate information about the victim could assist the police in their inquiries by helping to narrow the window of opportunity, facilitate more accurate reconstruction events, and aid in the gathering of evidence. Previous research demonstrates that changes occur to the structural composition of bone over both short and archaeological timescales.^22,24,25,37–41^ Alongside increases in the size and order of the mineral crystals, substitutions at the A-site, hydroxyl position, and the B-site, phosphate position, can result in changes to the carbonate content of the bone.^38,42–46^ Several studies have shown decreases in the carbonate–phosphate (C/P) ratio^39,41^ as a result of either carbonate loss, phosphate increase, or protein loss. An inverse relationship between crystallinity and C/P, where C/P decreases as crystallinity increases has been noted,^38,41,47^ and the loss of CO_3_^2–^ as a result of dissolution has also been observed.^38^ Protein loss is associated with collagen loss, increased porosity, and microbial attack.^38,39,41,47,48^ Bone exchanges elements with the deposition environment and changes to the elemental composition of bone are found in both archaeological contexts,^49–51^ and short timescale studies.^34,36,39,41,52,53^ Various elements have been studied as part of diagenesis research, particularly calcium and phosphate due to their presence in bone minerals. Iron, sodium, and potassium are related to bodily fluids, while other elements are present in trace amounts.^34,41^ A loss in sodium and potassium has been observed within 12 months PMI^34,41^ due to dehydration of the bone.^41^ A loss in iron has also been observed <12 months PMI and has been associated with the breakdown of blood cells.^34^ Studies have also noted changes to zinc,^52^ manganese, and magnesium^41^ occurring over time.

Bone trauma research has focused on the timing of trauma through the analysis of fracture characteristics.^54–56^ Distinguishing between fractures occurring in the perimortem and postmortem period is complicated as bone does not immediately show postmortem characteristics after death. Due to its porous nature, bone can retain moisture, collagen, and elasticity for weeks or months into the postmortem period.^54,55,57,58^ Any damage occurring while the bone still shows fresh characteristics can be misinterpreted as perimortem trauma despite occurring weeks or months into the postmortem period. Research on the timing of trauma has mainly focused on fracture characteristics and how and when these characteristics change.^54,55,59–61^ Distinguishing the time it takes for a bone to become “dry” is challenging as it is heavily influenced by the deposition environment.^55,58–61^ Initial research has investigated how fracture characteristics change over the postmortem period, focusing on how the taphonomic environment could obliterate evidence of trauma.^62,63^

Research has also focused on the physical aspects of the trauma; how fractures affect the macroscopic analysis of the bone and fracture morphology,^64–67^ with little focus on fractures and bone diagenesis. The research presented here aims to establish whether fractures to either fresh or dry bone can affect the physicochemical changes bones undergo post-deposition. As bone does not exhibit dry bone characteristics immediately after death,^54,55,59–61^ the terms perimortem and postmortem in relation to the timing of bone trauma can be ambiguous.^54^ For simplicity, the following definitions are used throughout this paper:

- Perimortem fractures: Damage occurred while the samples were fresh and displaying “wet bone” characteristics.^55,61^ Trauma was applied prior to deposition.
- Postmortem fractures: Samples were damaged 60 days after deposition. Samples were displaying “dry bone” characteristics.^55,61^

The overall hypothesis for this research was that there would be statistically significant differences between the physicochemical composition of the bone samples with (perimortem or postmortem) fractures compared to the unfractured bone samples (controls) after a diagenetic change had occurred. The null hypothesis was that no statistically significant differences would be observed between the two conditions (fractured versus non-fractured).

## Materials and Methods

These studies were conducted between September 2020 and May 2022 in the UK, with samples placed outside either in the summer, or winter seasons, and temperature data can be seen in Figure S1 (Supplemental Material). Excised pig (*Sus scrofa domesticus*) ribs (*n* = 149) were locally sourced, purchased fresh, and stored in a refrigerator overnight at 4 °C. Using a PM40 scalpel, the ribs were partially defleshed as close to the bone as possible without damaging the bone surface. Fresh defleshed pig ribs (*n* = 4) were saved to be used as day 0 controls. Details of the variables and timescales for this are shown in Table I.

**Table I.** Details of the variables/conditions included in this study, including the post-fracture intervals and the number of samples per variable.

Variable 1	Variable 2	Post-fracture interval (days)	Number of winter samples	Number of summer samples
Control	Control	0	4	
Perimortem	Control	30	4	5
		90	4	5
		180	4	5
	Blunt-force trauma	30	5	5
		90	5	5
		180	5	5
	Sharp-force trauma	30	5	5
		90	5	5
		180	5	5
Postmortem	Control	90	4	5
		180	4	5
	Blunt-force trauma	90	5	5
		180	5	5
	Sharp-force trauma	90	5	5
		180	5	5

### Perimortem Fractures

**Figure 1.**
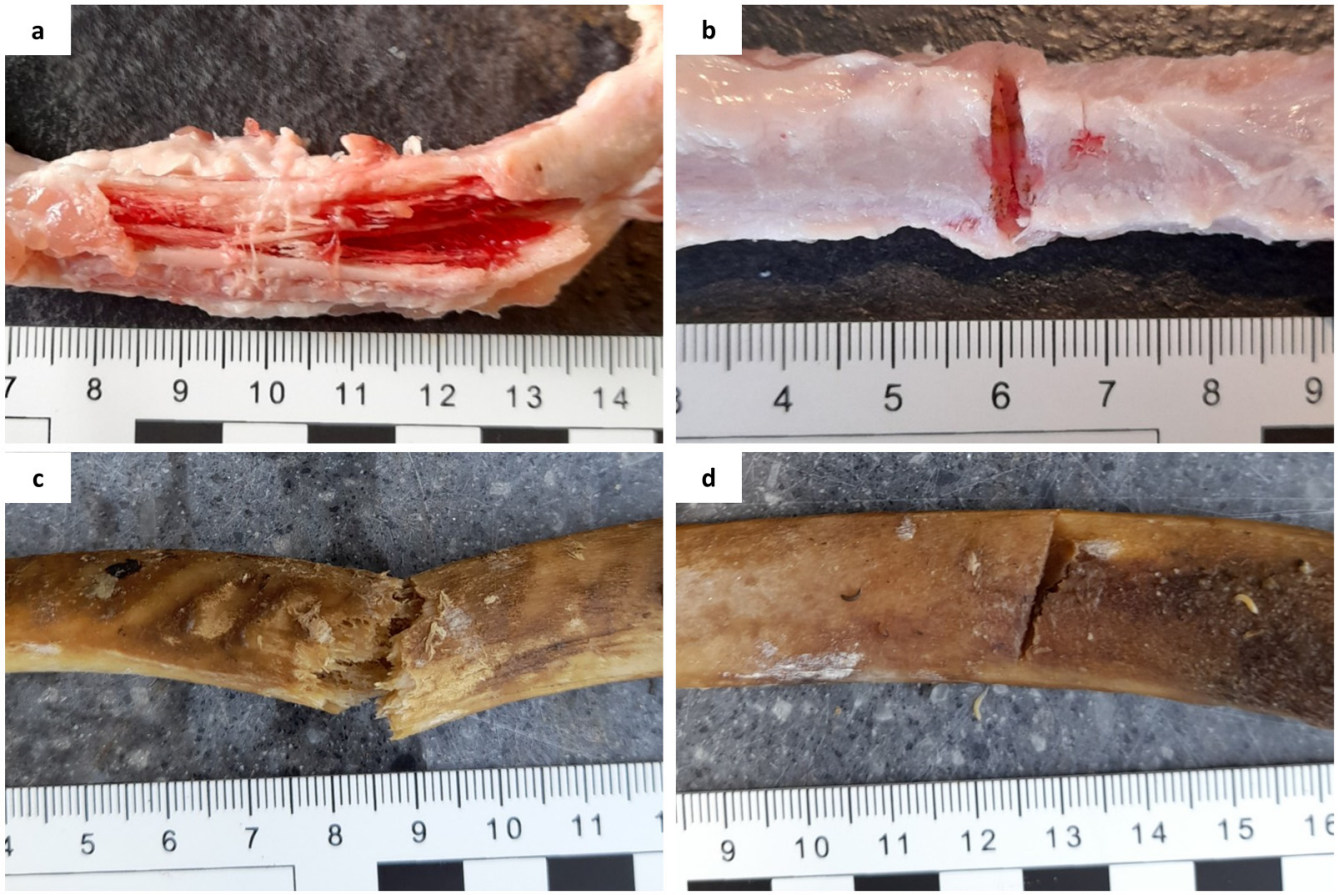
Freshly inflicted fractures on porcine rib bones: (a) perimortem blunt-force fractures, (b) perimortem sharp-force fractures, (c) postmortem (60 days PMI) blunt-force fractures, and (d) postmortem (60 days PMI) sharp-force fractures.

The samples were separated into control (*n* = 27) and experimental (*n* = 60) groups; blunt-force trauma (BFT) (*n* = 30) and SFT (*n* = 30). To create BFT, a small hammer (Rolson) attached to a purpose-built weapon holder with a 5 kg weight was dropped consistently from a height of 75 cm onto the samples. To create SFT, a small camping axe (Rolson) was attached to the weapon holder, with no extra weight, and dropped from a height of 45 cm onto the samples. Once damage had been inflicted, scale photographs were taken of the fractures (Figures 1a and 1b). All samples were deposited onto a patch of grass in a domestic garden in Huddersfield, West Yorkshire, UK, and left to decompose. Collection of samples from each of the three conditions (control, BFT, and SFT) took place at 30, 90, and 180 days after deposition.

### Postmortem Fractures

The remaining partially defleshed ribs (*n* = 58) were left exposed at the same deposition site as the perimortem samples to decompose for 60 days. After this time, 40 samples were collected for fracturing. The remaining samples were non-fracture controls (*n* = 18). For BFT, the small hammer was attached to the weapon holder, but no weight was added due to the fragility of the samples. Ten samples were hit with the hammer from a height of 75 cm. For the SFT, the remaining 10 samples were hit with the small camping axe, dropped from 45 cm high. Once fractures had been inflicted, photographs were taken (Figures 1c and 1d) and the samples were returned to the deposition site. Collection of samples from each of the three conditions took place at 90 and 180 days post-fracture (150 and 240 days PMI, respectively).

Perimortem samples (up to 90 days PMI) were macerated in freshwater at 40 °C as small amounts of tissue were still present. Samples were placed in padded bags for protection and lowered into a maceration pot with a Buffalo mechanical stirrer (DN868) for up to 90 min. High temperatures and added detergents were avoided as recommended in the literature.^2,9,54,68–70^ Samples were rinsed, dried, and frozen at −20 °C until analysis. The effect of freezing was considered,^71–73^ however, all samples were subject to the same storage conditions and therefore should be comparable. Note was taken of the conclusions by McElderry et al.^72^ and only one cycle of freezing and defrosting was undertaken.

### Physicochemical Analysis

A section of bone 3–5 mm thick was cut from every sample (from the middle of the control samples and at the fracture site for the experimental samples). The sections were dried overnight at 23 °C then stored at an ambient temperature of 23 °C until analysis.

Trace elemental analysis was conducted using a Jeol JCM 6000+ desktop SEM with an EDS analyzer (settings: filament current: high; probe current: high; accelerating voltage: 15 kV; working distance: 19 mm; magnification: 500×). Three elemental measurements of the outer edge of the cortical bone were taken per sample and averaged to give one overall reading per sample. Eight elements were selected using current literature^34,52^ as guidance; calcium and phosphorus to represent bone minerals, potassium, sodium, and iron to represent bodily fluids, and magnesium, zinc, and barium to represent trace elements. For analysis, the mass (%) of each selected element was calculated using analytical software in standardless quantitative mode.

**Figure 2.**
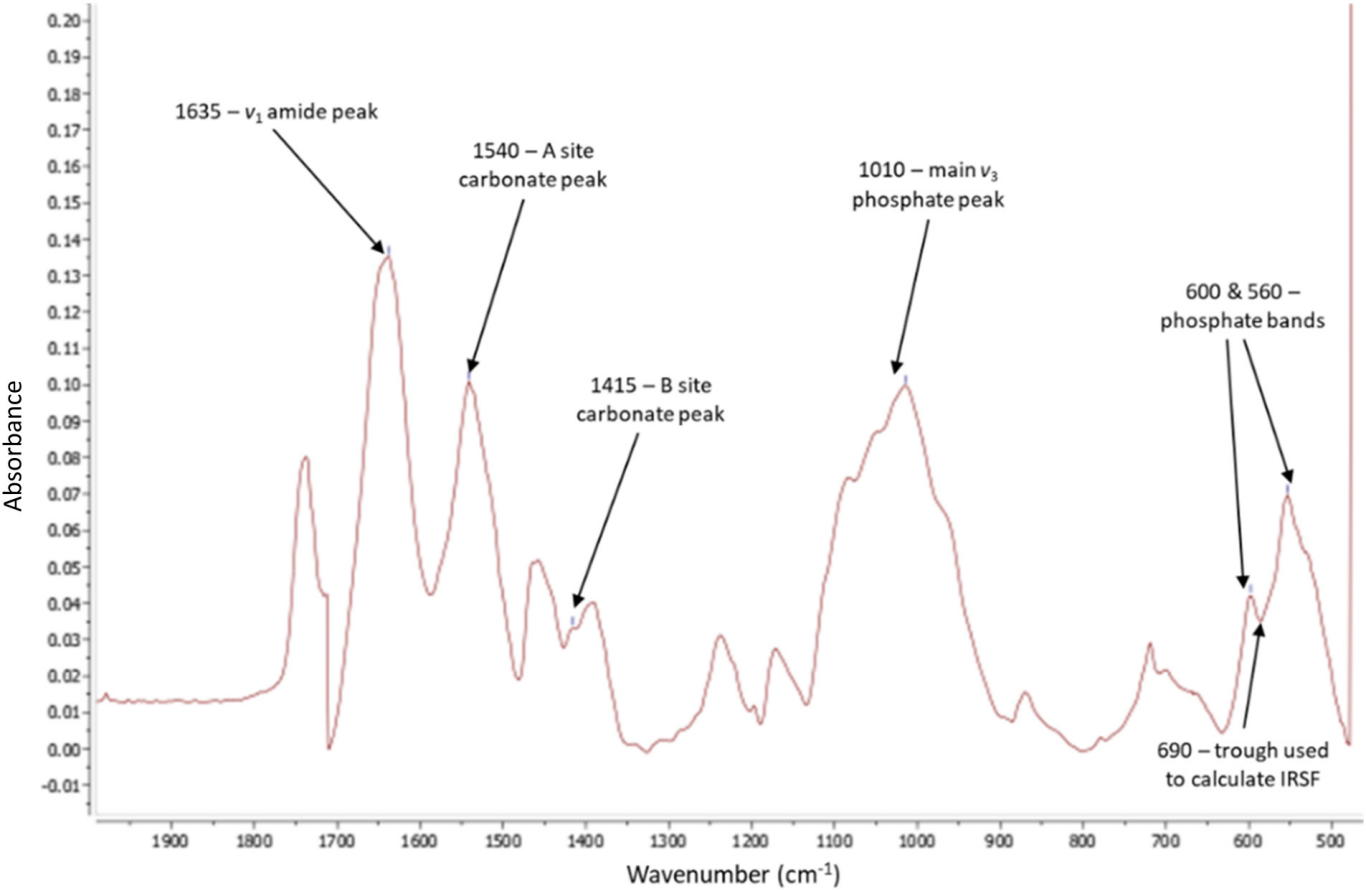
FT-IR spectra taken from MestReNova software. Arrows indicate the six peaks used for all calculations which bonds/functional groups they correspond to, and the trough used for IRSF. Baseline corrected at approximately 470 > 750 > 1250 > 1720.

To prepare for attenuated total reflection Fourier transform infrared spectroscopy (ATR FT-IR) analysis, the dried bone sections were broken into small fragments (approximately 1 mm in size) using a hammer, and the outer cortical bone fragments were collected. Measurements were conducted using a Thermo Scientific Nicolet iS5 FT-IR spectrometer fitted with an id7 ATR accessory with the following parameters range: 4000–400/450 cm^–^^1^; number of scans: 144; resolution: 4 cm^–^^1^; mode: absorbance. Bone fragments were placed onto the optic window to cover the diamond crystal and pressed onto the diamond using the pressure applicator. After each sample scan, the plate and pressure anvil were cleaned with ethanol. Six peaks were identified for analysis of the size and order of the mineral crystals using the infrared splitting factor (IRSF), carbonate, and protein content using protocols set out by Kontopoulos et al.,^42^ Sponheimer and Lee-Thorp,^74^ Trueman et al.,^75^ Weiner and Bar-Yosef,^76^ Wright and Schwarcz,^77^ and Hollund et al.^78^ (Figure 2 and Figure S2, Supplemental Material).

The background reading was measured before the start of each day and whenever a new set of samples was analyzed (per PMI), or every 100 min, whichever was sooner.

To analyze the data, the analytical chemistry software package, MestReNova was used. For each spectrum, the baseline was corrected, using Végh et al.^41^ as a guide, between 450 and 2000 cm^–1^ at approximately 470 > 650 > 800 > 1250 > 1750. The peak-by-peak function was used to obtain the heights of the required peaks while the manual threshold was used to determine the trough depth between peaks 560 and 600 cm^–1^ for the IRSF measurement. The protocol set out by Dal Sasso et al.^79^ was attempted to calculate the IRSF; however, the results were inconsistent for several samples, possibly due to the drawn baseline falling below 0 on the absorbance axis. The calculations used to establish changes to the mineral and amide content of the samples are shown in Table II. The calculations for measuring the amount of Type A and Type B carbonate relative to phosphate were done using the phosphate band at 1010 cm^–1^ using the protocol set out in Howes et al.^48^ The phosphate band at 600 cm^–1^ has been used in other research,^42,74^ however, the justification given by Howes et al.^48^ that the 1010 cm^–1^ peak could give more accurate results made it the better option.

**Table II.** Structural changes assessed using ATR FT-IR spectra.

Assessed changes	Description	Formulae for absorbance at peaks (cm^–1^)	References
Infrared splitting factor (IRSF)	Observe changes to the size and order of hydroxyapatite crystals within the mineral lattice	$\frac{600+560}{590}$	42,76–78
Type A carbonate–phosphate index (API)	Amount of Type A carbonate within the sample relative to phosphate	$\frac{1540}{1010}$	48
Type B carbonate–phosphate index (BPI)	Amount of Type B carbonate within the sample relative to phosphate	$\frac{1410}{1010}$	48
Type B-to-Type A index (BAI)	Amount of Type B carbonate to amount of Type A carbonate within the sample	$\frac{1410}{1540}$	74
Amide–phosphate ratio (Am/P)	Amount of protein within the sample relative to phosphate	$\frac{1640}{1010}$	42,75,78

### Statistical Analysis

The Shapiro–Wilkes test for normality was conducted on all data in the first instance to determine which statistical tests to undertake. As this showed the data was not normally distributed, non-parametric tests were performed. The Kruskal–Wallis test for independent samples was used alongside pairwise comparisons to show where significant differences, if found, were occurring. Although not the focus of this study the potential effect of the UK seasons could not be ignored, therefore the two data sets (winter and summer) were statistically analyzed separately to assess for any differences in significant results. Comparisons made for both elemental content and structural data were:

- Control samples versus all perimortem fractures
- Control samples versus perimortem BFT
- Control samples versus perimortem BFT
- Perimortem BFT versus perimortem SFT
- Control samples versus all postmortem fractures
- Control samples versus postmortem BFT
- Control samples versus postmortem BFT
- Postmortem BFT versus postmortem SFT
- Postmortem fractures versus perimortem fractures

## Results and Discussion

### Perimortem Fractures

**Figure 3.**
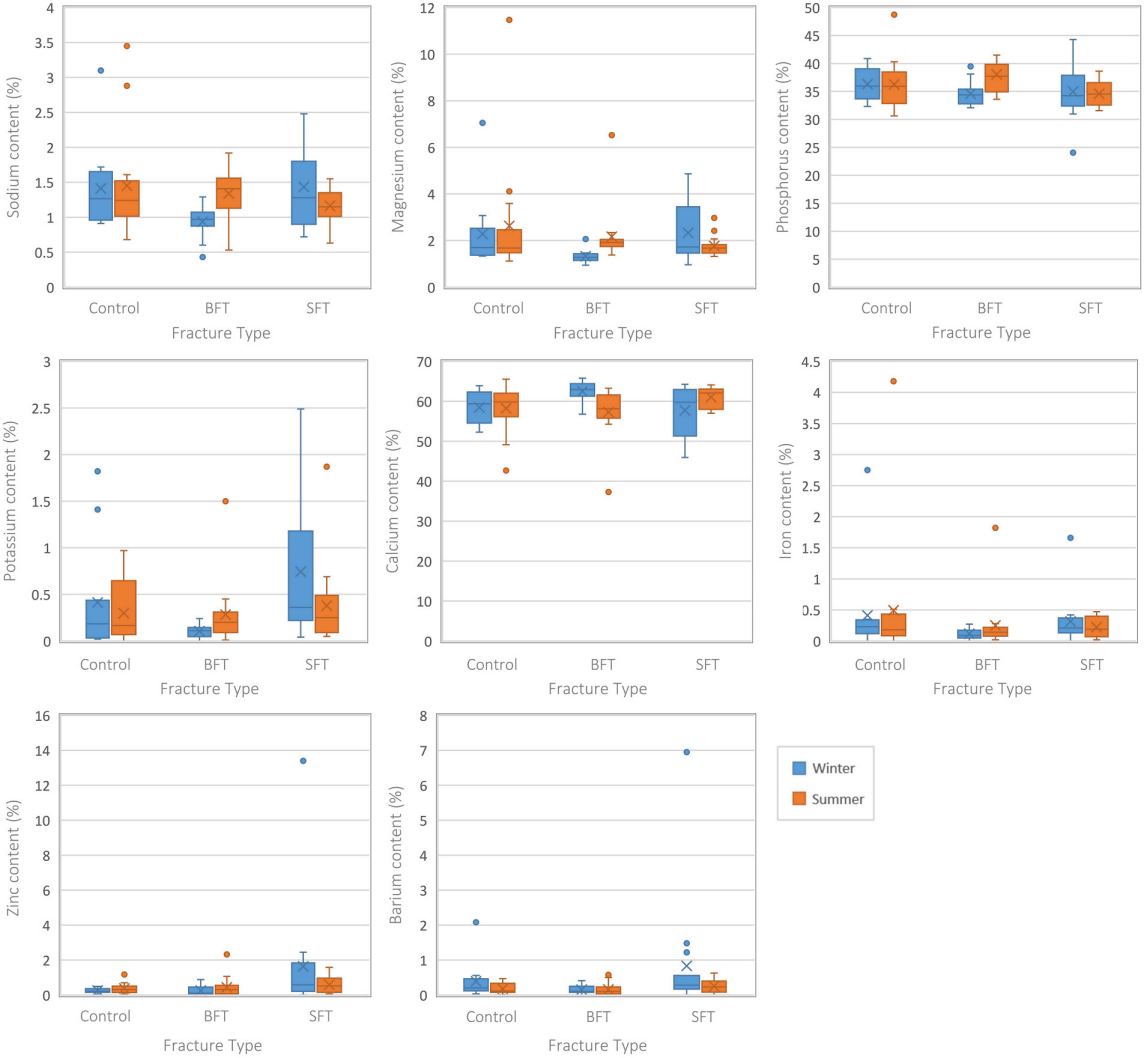
Boxplots representing the perimortem samples from both studies (all PMIs grouped together). Each plot represents a different element and the range of values for each variable (control, BFT, and SFT). Significant differences can be found in Table S1 (Supplemental Material).

Several differences were seen among the elemental composition of three conditions (control, BFT, and SFT), irrespective of time since trauma (Figure 3). Although there were variations in the data, no statistically significant differences were seen in the Ca and P contents among the three groups. The winter BFT samples showed lower levels of Na, K, Mg, Fe, and Zn than the control and SFT samples. The summer BFT samples did not completely agree with these results as they showed a higher Na content. Different elements were found to be statistically significant in the two data sets (Figure 3; Table S1, Supplemental Material); this would indicate that seasonality can play a role in diagenetically induced physicochemical change. Statistically significant (*p* < 0.05) differences (Table S1, Supplemental Material) were found among the three conditions for Ca, Na, K, Mg, Fe, and Zn in the winter samples, but the summer samples only showed P to be significant. Post-hoc tests showed these elements were all significantly different between the two fracture groups but only Na, Mg, and Ca of the winter samples were significantly different between the control and BFT groups. There were no significant differences between the control and SFT groups for either season.

Separating the samples by their post-fracture interval indicated at which timescale the differences occurred (Table S1, Supplemental Material). The winter BFT samples showed significantly (*p* < 0.05) lower levels of Na and Mg at 30 days post-fracture and lower levels of K at 90 days post-fracture, although post-hoc tests found this to be due to differences between the two fracture groups. Interestingly, higher levels of K in the SFT samples resulted in statistically significant (*p* < 0.05) differences between the SFT and control samples at 180 days post-fracture. Differences between the two fracture groups led to significant (*p* < 0.05) differences in Fe content for the winter samples at 180 days post-fracture.

**Figure 4.**
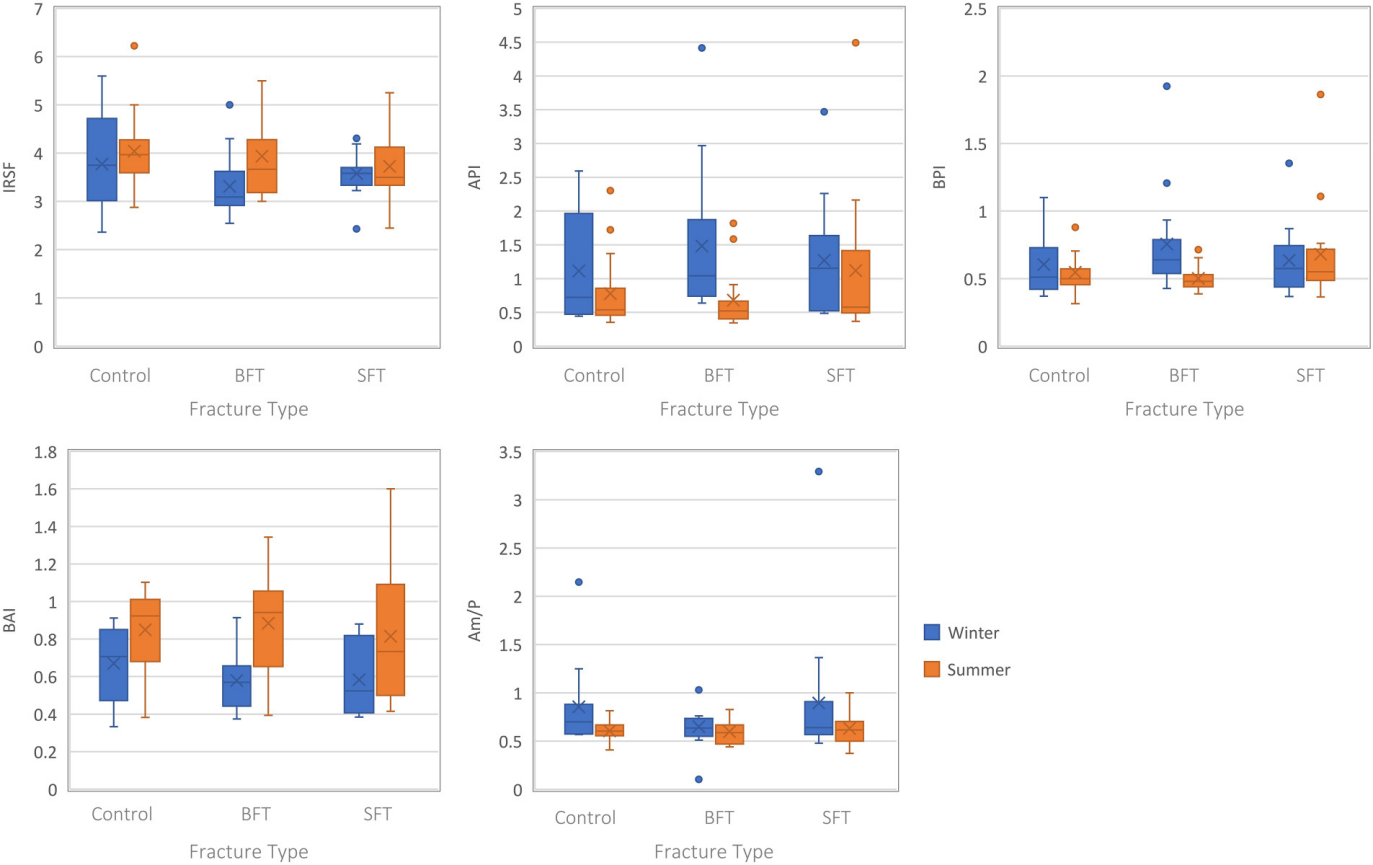
Boxplots representing the perimortem samples from both studies (all PMIs grouped together). Each plot represents a different IR parameter and the range of values for each variable (control, BFT, and SFT). Significant differences can be found in Table S1 (Supplemental Material).

Analysis of the structural composition of the samples from both winter and summer studies did not produce any statistically significant differences (*p *> 0.05) among the three groups (Table S1, Supplemental Material), although several infrared (IR) parameters were different for the summer samples when plotted as the Type A carbonate–phosphate index (API) and Type B carbonate–phosphate index (BPI) were higher in the SFT group (Figure 4).

Separating the samples by PMI showed significant differences among the three groups at 180 days PMI, however, this was only for the winter study (Table S1, Supplemental Material). The BPI was higher in the BFT group compared to the control and SFT groups. Post-hoc tests showed a significant difference in BPI between the control and BFT groups.

### Postmortem Fractures

**Figure 5.**
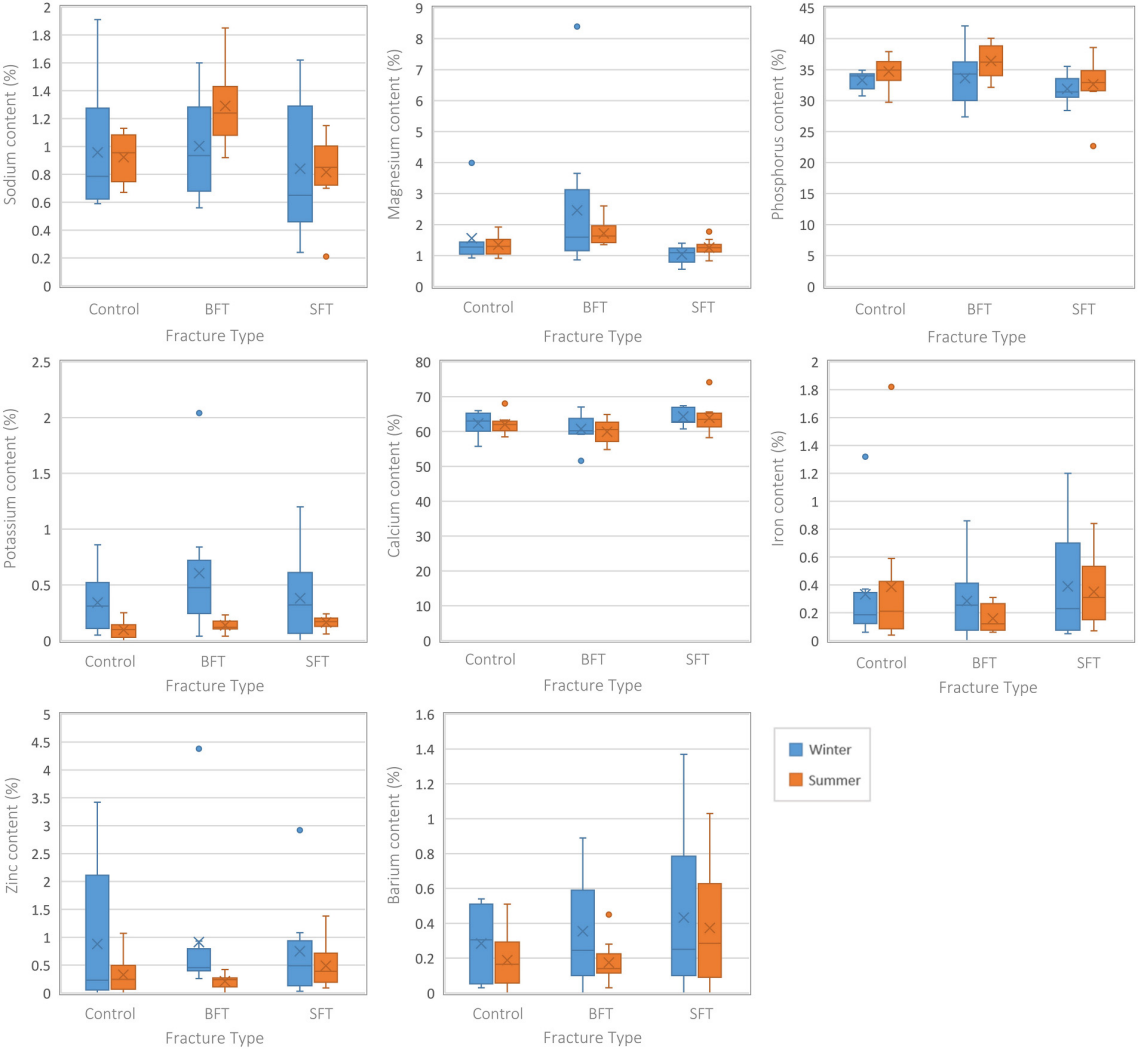
Boxplots representing the postmortem samples from both studies (all PMIs grouped together). Each plot represents a different element and the range of values for each variable (control, BFT, and SFT). Significant differences can be found in Table S2 (Supplemental Material).

Several differences were observed among the elemental composition of the three conditions (control, BFT, and SFT) (Figure 5), and differences were observed between both seasons. The winter BFT group had higher Mg and K levels, while the summer study showed higher levels of Na and Mg in the BFT group, and K content was lowest in the control group. Statistical testing (Table S2, Supplemental Material) showed Mg was significantly (*p *< 0.05) different among the three groups (control, BFT, and SFT) for both seasons, but post-hoc tests found this occurred between the two fracture groups. For the summer study, Na, Mg, and P were significantly (*p *< 0.05) different, and post-hoc tests indicated this was due to significant differences between the two fracture groups for all three elements. Further significant (*p *< 0.05) differences were found between the control and BFT groups for Na and Mg.

Separating the samples by their post-fracture interval also revealed statistically significant (*p *< 0.05) differences among the three groups for both seasons. At 90 days post-fracture, the winter study showed significant (*p *< 0.05) differences in the levels of Ca between the two fracture groups due to higher Ca content in the SFT group (Table S2, Supplemental Material). No statistically significant (*p *> 0.05) differences were found at 180 days post-fracture for this study. Although no statistically significant (*p *> 0.05) differences were found at 90 days post-fracture in the summer study, three elements (Na, P, and Ca) did display statistically significant (*p *< 0.05) differences at 180 days post-fracture. Na content was highest in the BFT group and lowest in the SFT group, while Ca content was highest in the SFT group and lowest in the BFT group. P content was highest in the BFT group. All three elements were significantly different between the two fracture groups in post-hoc testing (Table S2, Supplemental Material). Na was significantly (*p* < 0.05) different between the control and BFT groups, while Ca was significantly (*p* < 0.05) different between the control and SFT groups.

**Figure 6.**
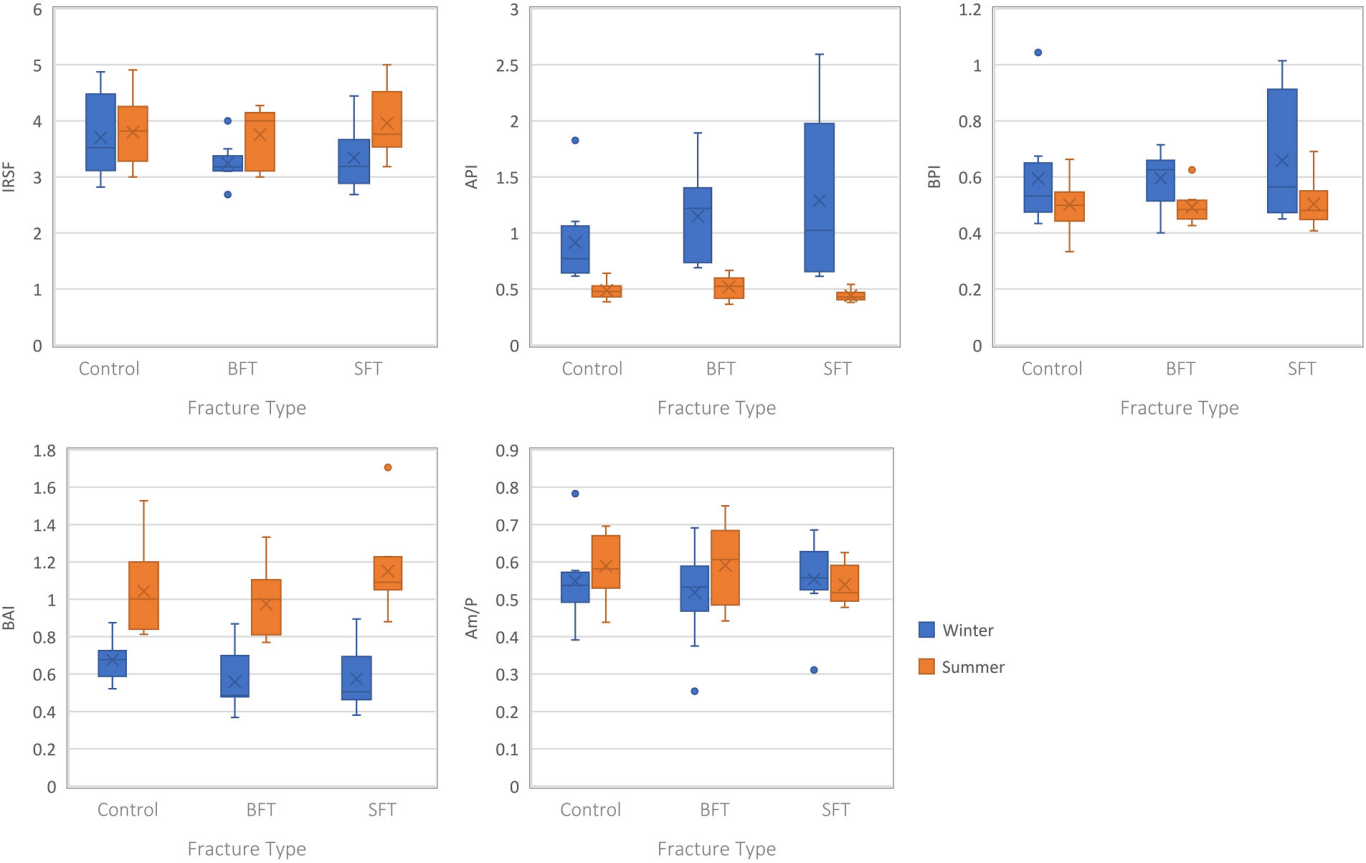
Boxplots representing the postmortem samples from both studies (all PMIs grouped together). Each plot represents a different IR parameter and the range of values for each variable (control, BFT, and SFT). Significant differences can be found in Table S2 (Supplemental Material).

No statistically significant (*p* > 0.05) differences were found in structural composition among the three conditions for either season (Table S2, Supplemental Material); however, variations in the data were observed when plotted (Figure 6). The winter study showed slightly higher crystallinity and Type B-to-Type A index (BAI) values in the control group, along with lower API and BPI values. Yet, the summer study showed higher crystallinity and BAI values in the SFT group, and slightly lower amide–phosphate ratio (Am/P) values.

Separating the samples by post-fracture interval did not yield any statistically significant results for the summer study (Table S2, Supplemental Material). However, at 180 days post-fracture multiple IR parameters were different for the winter study. Higher crystallinity values were observed for the control group compared to the two fracture groups, while the BFT group had higher API and lower BPI values. All three IR parameters were significantly (*p* < 0.05) different between the control and BFT groups in post-hoc tests, and the API was also significantly (*p *< 0.05) different between the two fracture groups.

### Perimortem Fractures Versus Postmortem Fractures

**Figure 7.**
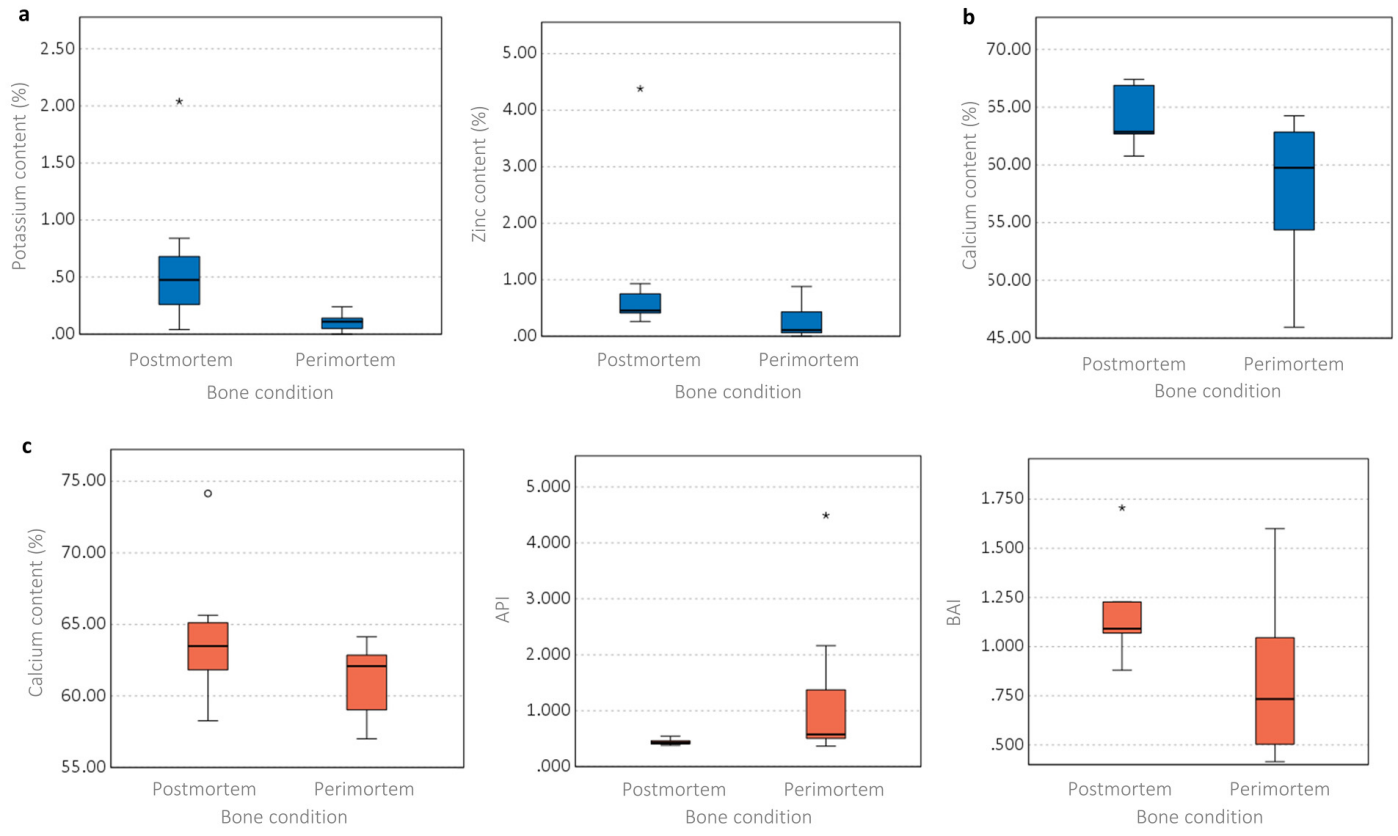
Boxplots representing the differences between the perimortem and postmortem conditions (blue: winter study; red: summer study). (a) Winter BFT samples, significant elemental content differences, (b) winter SFT samples, significant elemental content differences, (c) summer SFT samples, significant elemental content and structural differences. Significant differences among the parameters can be found in Table S3 (Supplemental Material).

Several elements were found to be significantly different between the perimortem and postmortem samples for both seasons (Figure 7 and Table S3, Supplemental Material). The winter study showed statistically significantly lower levels of K and Zn in the perimortem BFT samples (Figure 7a), which were not seen in the control and SFT samples. Ca was the only significantly different element for the winter SFT samples (Figure 7b), although this was also observed in the SFT samples of the summer study (Figure 7c). For both seasons, the perimortem samples had lower levels of Ca than the postmortem samples. Only the summer SFT samples showed any statistically significant differences for the ATR FT-IR analysis (Table S3, Supplemental Material). The API was higher in the perimortem samples, while the BAI was lower in these same samples (Figure 7c). The summer study did not show any statistically significant differences between the perimortem and postmortem samples for any IR parameter for the control of BFT samples (Table S3, Supplemental Material). As literature^80^ has suggested, the potential for inaccuracies in Type A carbonate calculations when using the 1540 cm^–1^ peak due to an overlap with amide II, the linear relationship between this peak and the amide I peak at 1640 cm^–1^ was assessed. This showed only weak relationships between the two peaks for both data sets (Figure S3, Supplemental Material).

### Perimortem Fractures

Several differences were observed among the physicochemical compositions of the control, BFT, and SFT samples indicating the presence of bone fractures can influence diagenetic change. Greater surface damage was visible on the BFT samples, with larger, deeper fractures present compared to the SFT samples (Figures 2a and 2b). Physical breakdown of the bone surface, i.e., fractures, is thought to influence the exchange of elements.^49^ Na and K are associated with bone dehydration.^34,41^ While all samples showed losses in Na and K compared to fresh bone samples, the winter study BFT samples experienced the biggest losses; this was particularly true for Na at 30 days post-fracture and K at 90 days post-fracture. This could indicate that the presence of extensive BFT fractures leads to increased moisture loss over time in the perimortem bones, however, it is also important that consideration is given to the deposition environment as the same differences were not observed in the summer study as the BFT samples from this study did not lose as much Na or K. It could be assumed that the summer samples would be most influenced by dehydration due to the dryer and warmer season, however, this was not the case here. These results could indicate that the loss of Na and K is not primarily driven by the loss of moisture, but possibly by the movement of water within the bone. It is suggested that the winter BFT samples suffered the biggest losses in Na and K due to the extensive fractures aiding in the easier movement of rainwater through the bone and “flushing” out the elements. This theory can be supported by the lack of elemental exchange seen in the control samples, which had no surface damage. Similarities between the control and SFT samples could be due to the relative lack of surface damage present on many of the SFT samples, which in many cases did not breach the full cortical thickness, although this would not explain the apparent increase in K in the winter SFT samples at 180 days post-fracture. Although significant differences were not observed among the three groups at 90 or 180 days post-fracture, this is consistent with current literature,^41^ which suggests the loss of Na slows after an initial loss at <30 days PMI.

Slight losses in Mg and Fe content occurred in all three groups and across both studies when compared to the fresh bone. Both seasonal studies saw the BFT samples lose the most Fe compared to the control and SFT groups, while the winter BFT samples were the most affected by Mg loss. As Fe is associated with blood,^34,41^ it is likely that the considerable loss of Fe in the BFT samples was caused by hemolytic breakdown facilitated by the extensive fractures, caused by the mini hammer. This may also explain the negligible Fe loss observed in the control and SFT samples, which had little, or no, damage to the bone surface. This was particularly noticeable at 180 days post-fracture when the winter study showed statistically significant differences in the Fe content of the two fracture groups. Mg is a trace element typically found within both bone and soil, and Mg levels have been shown to be affected by microbial action.^81^ The lower elemental levels seen in the winter BFT samples may be due to the damaged bone surface facilitating the passage of water, or the action of microbes, thus increasing the exchange of ions between the bone and the soil. This is supported by an observed absence of elemental exchange seen in the control samples, which had no surface damage; and the SFT samples which only incurred minor surface damage that did not breach the full cortical thickness. It has been shown that groundwater can affect bone diagenesis,^82^ therefore it is possible that the bone damage at the BFT fracture sites allowed water from the deposition environment to pass through the bone influencing the uptake and loss of several elements. As the winter samples were exposed to a wetter environment, the increased presence of water may have exacerbated this loss of elements compared to the samples from the summer study. Zn levels decreased slightly in the control and BFT samples for both studies, while an unexpected increase was observed in the winter SFT samples. This resulted in statistically significant differences between the three groups (*p* < 0.05) for the winter study. It is unclear why the SFT samples increased in Zn, and this needs more investigation.

While the winter BFT samples showed slightly elevated Ca levels, these were comparable with fresh, day 0 samples, and the remaining samples all showed slight losses in Ca content. Several studies^34,41^ have shown Ca to remain relatively stable in the postmortem period. Keenan and Engel^39^ did note losses in Ca in alligator bones due to the incorporation of Fe over time; however, their samples also showed signs of bioerosion which may have been an influencing factor for these changes. The actions of microbes have been shown to affect the physicochemical composition of postmortem bone,^14,39,81,83^ therefore it is possible the changes observed this this study were influenced by bioerosion; however, this was not assessed. Furthermore, it is noted that scanning electron microscopy energy dispersive spectroscopy (SEM-EDS) does not give absolute measurements, but instead determines a percentage mass based on the elements isolated.^84^ As the measurements given are relative, a significant change in one (or more) element could potentially affect other elements. P was the only element to show a statistically significant difference among the three groups for the summer study and this was due to bigger increases in the BFT samples compared to the control and SFT samples. P has been shown to stay relatively stable throughout the postmortem period in other studies,^34,39,41^ although this was found on samples without fractures. The increases in P observed here may have been due to the presence of fractures, but this would not explain the slight increases also seen in the control and SFT samples.

There were no statistically significant differences in any IR parameters among the three conditions for either seasonal study. Considering the differences observed in the elemental analysis, this was unexpected and would indicate that the presence of perimortem fractures did not have a significant influence on the structural composition of the bone. Although not statistically significant, variations in the data were observed in the summer study for several IR parameters (Figure 4). The summer SFT samples appeared to show higher carbonate content, as measured through the API and BPI, compared to the control and BFT samples.

Separating the samples by their post-fracture interval still did not yield any significant differences among the three groups for the summer study, however, one IR parameter was significantly different in the winter study at 180 days post-fracture. At this timescale, the BFT samples showed a higher BPI than the control and SFT samples. All three groups showed increases in Type B carbonate content compared with fresh samples, but the BFT was the most affected. This indicates that the presence of perimortem fractures can affect the structural composition of bone, but not as quickly as it causes elemental changes. It may be possible that the presence of BFT influenced the results observed at 180 days PMI, as increased Type B carbonate substitutions occurred in the BFT samples at the expense of phosphate.

### Postmortem Fractures

Several physicochemical differences were observed among the three conditions (control, BFT, and SFT). However, Mg was the only element to be affected by the presence of postmortem fractures in both seasonal studies. Both studies saw increases in Mg levels for the BFT samples while the control and SFT samples had slight losses compared to fresh bone. As the BFT samples showed increases in Mg content, it is likely the extensive fractures (Figures 2c and 2d) influenced ion exchange through bone–soil interactions at the fracture sites. These changes in Mg contrast with those observed in the perimortem samples, where a loss in Mg was seen. It is likely that the condition of the bones at the time of fracturing may have influenced the differences seen. Whilst previous studies have shown that Mg is hardly affected by diagenesis in undamaged samples,^34^ the samples in our current study were damaged postmortem, which may explain why they show a different trend. The fresh (wet) condition of the perimortem BFT samples may have influenced an increased loss in Mg, via extensive fractures as the bones dehydrated and succumbed to diagenesis. However, the postmortem samples may have been differently influenced due to their dry condition at the time of fracturing, and slightly increased deposition period (up to 240 days overall). It has been suggested that bone cracks could allow the deposition of exogenous material into the bone,^20^ so Mg, a trace element found in soil, may have been deposited during the transport of water or microbial action, through the dry, and already diagenetically altered, bone.

Although the Na content of the three groups for the winter study did not show any significant differences, the summer study did show statistically significant results overall, and again at 180 days post-fracture. The BFT samples showed higher levels of Na than the control and SFT samples however, upon comparing these results with the Na content of fresh bone, it was found that the BFT samples had Na levels consistent with the fresh bone, and the control and SFT samples had lost Na. As with Mg, differences in Na levels were observed between the perimortem and postmortem data sets as the perimortem BFT samples showed a loss in Na throughout the study which was not replicated in the postmortem BFT samples. Although the extended depositional period of the postmortem samples cannot be ruled out as an influencing factor here, it is likely that the differences seen between the two data sets were due to the condition of the bones at the time of fracturing.

Variations in the data were seen in the K content for both studies as all groups saw losses in K compared to fresh bone. However, the two seasons appeared to influence the bones in different ways as the winter study showed slightly higher K content in the BFT samples while the control and SFT samples had similar levels, yet the summer study showed lower levels in the control samples compared to the two fracture groups (Figure 5). These differences could have occurred because of the environment they were exposed to during the studies and indicate that different UK seasons can influence diagenetic alterations and is an area that requires further exploration. The winter study was conducted in a colder, wetter environment which may have inhibited moisture loss, it is possible water became trapped within the BFT fractures and further inhibited moisture loss compared to the control and SFT samples. Differences among the control samples and the two fracture groups for the summer study could be due to the presence of bone fractures. It is possible the fractures (BFT and SFT) allowed water to become trapped within the pores of the cortical bone and inhibited the loss of moisture, while the control samples, having no surface damage, did not undergo the same issue. Although the summer study shows more subtle differences among the three groups compared to the winter study, this could be due to the different seasons they were exposed to as the summer samples will have been exposed to less rainfall and therefore had less water available to become trapped. The postmortem samples showed a much greater loss in K than the perimortem samples, but this could be explained by the longer depositional interval of the postmortem samples.

Some samples showed slight increases in Ca and P in the postmortem samples, but the majority of samples were consistent with the Ca and P levels of fresh bone; both elements have been found to remain stable over increasing PMIs.^34,41^ However, these slight increases in P did lead to significant differences between the two fracture groups for the summer study, both overall and at 180 days post-fracture as the BFT samples showed higher increases than the SFT samples. The winter study showed different Ca content between the two fracture groups at 90 days post-fracture due to increases in Ca in the SFT samples. The summer study took longer to show significant differences, at 180 days post-fracture the SFT samples had increased in Ca content which led to significant differences among the three groups.

There were no statistically significant differences in any IR parameter among the three groups for either seasonal study, although some variations in the data were observed (Figure 6). Crystallinity and BAI increased in all samples compared to fresh bone, but the winter control samples and the summer SFT samples showed the biggest increases. Although the summer BFT samples showed bigger crystallinity changes than the winter BFT samples, they were still less affected overall than the control and SFT samples. Increases in crystallinity indicate changes in the size and order of the crystals within the bioapatite. It has been proposed that crystallinity changes occur due to an influx of water from the deposition site into the pores of the bone which then evaporates near the bone surface,^75^ however, if this were the case here, bigger changes in the BFT samples should have occurred due to increased water absorption through the extensive fractures. Instead, in this study, the opposite has occurred; this could indicate the presence of extensive fractures that allow water to pass more easily through the bone resulting in reduced changes to the size and order of the crystals. The BAI changes could indicate increases in type B carbonate alongside losses in type A carbonate. Further carbonate content differences were noted among the three groups from the winter study as lower API and BPI values were observed in the control samples. Interestingly, upon comparing these results with fresh bone, it was found that the API of the control samples was in line with fresh bone values and the two fracture groups had increased, while the BPI of the winter samples increased for all three groups. Although the API increased in the fracture samples, this could be explained by a loss in P; the Type B carbonate may be substituting the phosphate molecule resulting in the BPI increase and the loss in phosphate leading to the observed API increase.

The summer study showed variations in the amide content of the three groups. The SFT samples showed the biggest loss in protein content, although it is unclear why this occurred as it had been predicted that the BFT samples would be more susceptible due to the extensive fractures as seen with the elemental results.

As with the perimortem samples, statistically significant differences did not occur until 180 days post-fracture and were only seen in the winter study. At this interval, the IRSF, API, and BPI were affected. Crystallinity increased the most in the control samples compared to the two fracture groups, while higher API and BPI values were found for the BFT samples. All three parameters were significantly different between the control and BFT groups. This would indicate that the presence of extensive fractures could influence the structural composition of the bioapatite, but this effect was not seen at <180 days post-fracture in this study.

### Perimortem Fractures Versus Postmortem Fractures

One of the main themes of existing bone trauma/fracture research has been the search for methods to establish the timing of fractures, i.e., whether they occurred during the perimortem period or are the result of taphonomic damage. Due to the complexities of bone degradation, this has not proven easy.

Several elements were different between the perimortem and postmortem samples for both seasons (Figure 7). The winter study showed the perimortem BFT samples were statistically significantly lower in K and Zn content than the postmortem BFT samples. Losses in K content were observed in both the postmortem and perimortem BFT groups compared to fresh bone, however, the perimortem samples were susceptible to the greatest loss. The Zn content of the postmortem samples was in line with fresh bone while slight decreases in Zn were observed in the perimortem samples. This could indicate that the presence of extensive fractures occurring when a bone is still “fresh” could influence bone–soil interactions and ion exchange. As these differences were not observed in the summer study, it is theorized that the colder, wetter weather aided the results observed here, as the increased availability of water through rainfall may have increased the transport of water through the cortical bone, resulting in the leeching of elements. Ca was the only element to be significantly affected in both seasons, although only for the SFT samples. In both studies, lower Ca levels were found for the perimortem samples compared to the postmortem samples. For the winter study, both postmortem and perimortem samples lost Ca, however, the summer study only saw losses for the perimortem samples. Ca makes up a significant portion of the hydroxyapatite. Walden et al.^34^ found the hydroxyapatite in undamaged bone to be minimally affected in short timescales, while Keenan and Engel^39^ observed losses in Ca over time in alligator bone which they attributed to microbial action. It may be that microbial action was the influencing factor in the Ca changes observed here, but further research would be needed to confirm this. These results indicate the importance of the presence of perimortem and postmortem fractures on the mineral lattice. However, it is unclear why the SFT samples were affected rather than the BFT samples which exhibited more complex fractures, or the control samples which had no visible surface damage.

The ATR FT-IR analysis revealed some differences between the perimortem and postmortem samples, but only for the SFT samples of the summer study. The carbonate content was found to be affected as the API was lower in the postmortem samples compared to the perimortem samples, while the BAI was higher in the postmortem samples. The postmortem samples had API values such as fresh bone but slight increases in API occurred in the perimortem samples, but the BAI increased for both conditions. Decreases in carbonate–phosphate ratios have been noted in the literature^39,41^ due to either carbonate loss, phosphate increase, or protein loss, but a slight increase was observed here; this could indicate either carbonate increase or phosphate loss. A change in protein may be influential but the Am/P did not produce any significant difference between the two groups. Increases in BAI can occur due to increases in type B carbonate and/or decreases in type A carbonate. These observed differences between perimortem and postmortem fractures could indicate that SFT fractures occurring to either fresh or dry bone can influence the exchange of the hydroxyl and phosphate groups. The literature has suggested that using the peak at 1540 cm^–1^ for assessing type A carbonate content can lead to unreliable data due to an overlap with the amide II peak at this wavenumber.^80^ However, an analysis of the correlations between the peaks at 1540 and 1640 cm^–1^ showed weak linear relationships between the two (Figure S3, Supplemental Material). While a strong correlation would suggest the 1540 cm^–1^ peak was likely a result of amide II rather than type A carbonate, a weak relationship suggests this peak is type A carbonate. Statistical testing showed significant differences for API but not Am/P (Table S3, Supplemental Material). Although it cannot be ruled out that the 1540 cm^–1^ peak may be due to amide II, the lack of significant results for Am/P content suggests this is not the case here.

## Conclusion

This was a novel approach to investigate how the presence of bone fractures could influence the extent of diagenetic change in short timescales. Several elements were found to be significantly affected by the presence of fractures (both perimortem and postmortem), but the structural composition was unaffected until 180 days post-fracture, suggesting that bones are quickly susceptible to elemental changes but are structurally robust in the early postmortem period (<180 days). Significant differences were found in the physicochemical composition between the bones fractured perimortem and those fractured postmortems. Notably, the SFT samples appeared to be most influenced by the condition of the bone at the time of fracturing as differences were observed for both the elemental and structural composition of the bone, while for the BFT samples only elemental differences were found. Several parameters, including K, Zn, Ca, and the carbonate content, were found to be significant in distinguishing between perimortem and postmortem fractures, and require further investigation.

It has been shown here that SEM-EDS can be used to identify bone fractures and that, when used in conjunction with ATR FT-IR, has the potential to distinguish between perimortem and postmortem fractures. However, as this study only considered an exposed deposition and limited timescales, more research is needed. Not only did these techniques establish that the presence of bone fractures can influence the physicochemical composition of bone, but it was also possible to distinguish between the two types of fractures through the analysis of several elements and structural parameters (Table S3, Supplemental Material). This study deliberately focused on non-destructive analytical techniques to promote their use in forensic anthropology and bioarchaeology. Both SEM-EDS and ATR FT-IR provide quick, easy-to-use techniques that allow quantification of the composition of the bone and can provide preliminary data before destructive analysis is undertaken.

This study has shown that the presence of bone fractures can affect the diagenetic process. It has important applications in forensic anthropology for not only the prediction of the presence of perimortem fractures from diagenetic bone, but also for estimating the relative timing of fracturing, whether perimortem or postmortem; as well as the differentiation between perimortem and postmortem fractures. This could aid reconstruction of the events leading to death and assist with the sequencing of fractures that have occurred since death. This has applications in both forensic and archaeological contexts, where remains may have been deliberately or accidentally damaged after death, possibly to conceal perimortem trauma, for example in mass grave situations where bodies may have been moved after initial deposition, or where remains have been damaged by agricultural machinery. In archaeological contexts, this research could aid the prediction and reconstruction of the condition of remains in the early postmortem and taphonomic periods.

## Supplemental Material

sj-pdf-1-asp-10.1177_00037028231213889 - Supplemental material for Recognition of the Presence of Bone Fractures Through Physicochemical Changes in Diagenetic Bone Click here for additional data file. Supplemental material, sj-pdf-1-asp-10.1177_00037028231213889 for Recognition of the Presence of Bone Fractures Through Physicochemical Changes in Diagenetic Bone by Caley Mein, Jennifer R. Jones, Catherine Tennick and Anna Williams in Applied Spectroscopy
