# Supplementary material for: Recognition of the Presence of Bone Fractures Through Physicochemical Changes in Diagenetic Bone
Source: Appl Spectrosc. 2023 Nov 13;78(2):159–74. doi: 10.1177/00037028231213889 (PMC10832324; doi:10.1177/00037028231213889)
Supplement: sj-pdf-1-asp-10.1177_00037028231213889 - Supplemental material for Recognition of the Presence of Bone Fractures Through Physicochemical Changes in Diagenetic Bone [file sj-pdf-1-asp-10.1177_00037028231213889.pdf]

Supplementary Material

Recognition of the presence of bone fractures through physicochemical changes in diagenetic bone

Mein, C*; Jones, J.R; Tennick, C; Williams, A.

Research Centre for Field Archaeology and Forensic Taphonomy, School of Law & Policing, University of Central Lancashire, Preston, PR1 2HE, UK

*Corresponding author

Email address: cmein@uclan.ac.uk

[Contents]:

Figure S1: Temperature data

Table S1: Complete statistical data for the physicochemical analysis of the perimortem fracture samples (control vs fracture samples)

Table S2: Complete statistical data for the physicochemical analysis of the postmortem fracture samples (control vs fracture samples)

Figure S2: FTIR sample spectra from the winter study

Figure S3: Scatterplots for the 1540cm^{-1} and 1640cm^{-1} correlations

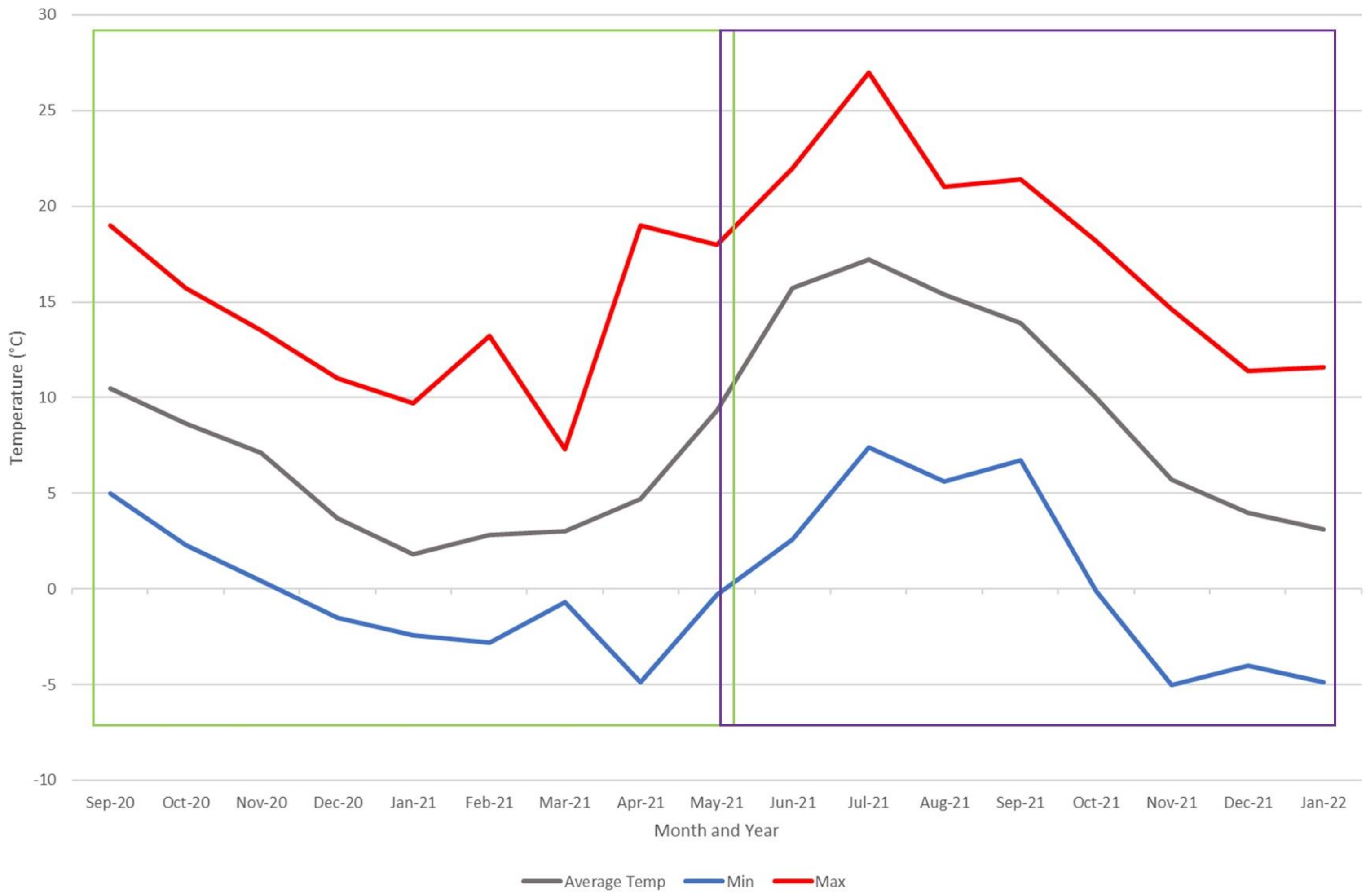

Figure S1 Temperature data for the two studies. The winter study was conducted between September '20 – May '21 (green box), the summer study was conducted between May '21 – Jan '22 (purple box).

Table S1 Complete statistical data for the physicochemical analysis of the perimortem fracture samples (control vs fracture samples)

	Grouping Variables	Dependant Variable												
		Na	Mg	P	K	Ca	Fe	Zn	Ba	IRSF	API	BPI	BAI	Am/P
Winter Experiment	Fracture vs control – all (n=41)	**0.017**	**0.003**	ns	**0.002**	**0.022**	**0.048**	**0.025**	ns	ns	ns	ns	ns	ns
	Control vs BFT	**0.015**	**0.011**	ns	ns	**0.016**	ns	ns	ns	ns	ns	ns	ns	ns
	Control vs SFT	ns	ns	ns	ns	ns	ns	ns	ns	ns	ns	ns	ns	ns
	BFT vs SFT	**0.013**	**0.001**	ns	**<0.001**	**0.019**	**0.021**	**0.01**	ns	ns	ns	ns	ns	ns
	Fracture vs control – 30 days PMI (n=14)	**0.02**	**0.04**	ns	ns	ns	ns	ns	ns	ns	ns	ns	ns	ns
	Control vs BFT	ns	ns	ns	ns	ns	ns	ns	ns	ns	ns	ns	ns	ns
	Control vs SFT	ns	ns	ns	ns	ns	ns	ns	ns	ns	ns	ns	ns	ns
	BFT vs SFT	**0.005**	**0.011**	ns	ns	ns	ns	ns	ns	ns	ns	ns	ns	ns
	Fracture vs control – 90 days PMI (n=13)	ns	ns	ns	**0.02**	ns	ns	ns	ns	ns	ns	ns	ns	ns
	Control vs BFT	ns	ns	ns	ns	ns	ns	ns	ns	ns	ns	ns	ns	ns
	Control vs SFT	ns	ns	ns	ns	ns	ns	ns	ns	ns	ns	ns	ns	ns
	BFT vs SFT	ns	ns	ns	**0.006**	ns	ns	ns	ns	ns	ns	ns	ns	ns
	Fracture vs control – 180 days PMI (n=14)	ns	ns	**0.038**	**0.037**	ns	**0.034**	ns	ns	ns	ns	**0.029**	ns	ns
	Control vs BFT	ns	ns	**0.019**	ns	ns	ns	ns	ns	ns	ns	**0.009**	ns	ns
	Control vs SFT	ns	ns	**0.032**	**0.013**	ns	ns	ns	ns	ns	ns	ns	ns	ns
	BFT vs SFT	ns	ns	ns	ns	ns	**0.01**	ns	ns	ns	ns	ns	ns	ns
Summer Experiment	vs control – all (n=44)	ns	ns	**0.03**	ns	ns	ns	ns	ns	ns	ns	ns	ns	ns
	Control vs BFT	ns	ns	ns	ns	ns	ns	ns	ns	ns	ns	ns	ns	ns
	Control vs SFT	ns	ns	ns	ns	ns	ns	ns	ns	ns	ns	ns	ns	ns
	BFT vs SFT	ns	ns	**0.009**	ns	ns	ns	ns	ns	ns	ns	ns	ns	ns
	Fracture vs control – 30 days PMI (n=15)	ns	ns	ns	ns	ns	ns	ns	ns	ns	ns	ns	ns	ns
	Control vs BFT	ns	ns	ns	ns	ns	ns	ns	ns	ns	ns	ns	ns	ns
	Control vs SFT	ns	ns	ns	ns	ns	ns	ns	ns	ns	ns	ns	ns	ns
	BFT vs SFT	ns	ns	ns	ns	ns	ns	ns	ns	ns	ns	ns	ns	ns

	Grouping Variables	Dependant Variable												
		**Na**	**Mg**	**P**	**K**	**Ca**	**Fe**	**Zn**	**Ba**	**IRSF**	**API**	**BPI**	**BAI**	**Am/P**
	**Fracture vs control – 90 days PMI (n=15)**	ns	ns	ns	ns	ns	**0.03**	ns	ns	ns	ns	ns	ns	ns
	**Control vs BFT**	ns	ns	ns	ns	ns	**0.012**	ns	ns	ns	ns	ns	ns	ns
	**Control vs SFT**	ns	ns	ns	ns	ns	ns	ns	ns	ns	ns	ns	ns	ns
	**BFT vs SFT**	ns	ns	ns	ns	ns	ns	ns	ns	ns	ns	ns	ns	ns
	**Fracture vs control – 180 days PMI (n=14)**	ns	ns	ns	ns	ns	ns	ns	ns	ns	ns	ns	ns	ns
	**Control vs BFT**	ns	ns	ns	ns	ns	ns	ns	ns	ns	ns	ns	ns	ns
	**Control vs SFT**	ns	ns	ns	ns	ns	ns	ns	ns	ns	ns	ns	ns	ns
	**BFT vs SFT**	ns	ns	ns	ns	ns	ns	ns	ns	ns	ns	ns	ns	ns

Table S2 Complete statistical data of the physicochemical analysis of the postmortem fracture samples (control vs fracture samples)

	Grouping Variables	Dependant Variable												
		Na	Mg	P	K	Ca	Fe	Zn	Ba	IRSF	API	BPI	BAI	Am/P
Winter Experiment	Fracture vs control – all (n=27)	ns	**0.04**	ns	ns	ns	ns	ns	ns	ns	ns	ns	ns	ns
	Control vs BFT	ns	ns	ns	ns	ns	ns	ns	ns	ns	ns	ns	ns	ns
	Control vs SFT	ns	ns	ns	ns	ns	ns	ns	ns	ns	ns	ns	ns	ns
	BFT vs SFT	ns	**0.011**	ns	ns	ns	ns	ns	ns	ns	ns	ns	ns	ns
	Fracture vs control – 90 days post-fracture (n=14)	ns	ns	ns	ns	**0.049**	ns	ns	ns	ns	ns	ns	ns	ns
	Control vs BFT	ns	ns	ns	ns	ns	ns	ns	ns	ns	ns	ns	ns	ns
	Control vs SFT	ns	ns	ns	ns	ns	ns	ns	ns	ns	ns	ns	ns	ns
	BFT vs SFT	ns	ns	ns	ns	**0.016**	ns	ns	ns	ns	ns	ns	ns	ns
	Fracture vs control – 180 days post-fracture (n=13)	ns	ns	ns	ns	ns	ns	ns	ns	**0.036**	**0.044**	ns	**0.037**	ns
	Control vs BFT	ns	ns	ns	ns	ns	ns	ns	ns	**0.01**	**0.024**	ns	**0.014**	ns
	Control vs SFT	ns	ns	ns	ns	ns	ns	ns	ns	ns	ns	ns	ns	ns
	BFT vs SFT	ns	ns	ns	ns	ns	ns	ns	ns	ns	**0.049**	ns	ns	ns
Summer Experiment	Fracture vs control – all (n=29)	**0.002**	**0.007**	**0.046**	ns	ns	ns	ns	ns	ns	ns	ns	ns	ns
	Control vs BFT	**0.006**	**0.026**	ns	ns	ns	ns	ns	ns	ns	ns	ns	ns	ns
	Control vs SFT	ns	ns	ns	ns	ns	ns	ns	ns	ns	ns	ns	ns	ns
	BFT vs SFT	**<0.001**	**0.002**	**0.013**	ns	ns	ns	ns	ns	ns	ns	ns	ns	ns
	Fracture vs control – 90 days post-fracture (n=15)	ns	ns	ns	ns	ns	ns	ns	ns	ns	ns	ns	ns	ns
	Control vs BFT	ns	ns	ns	ns	ns	ns	ns	ns	ns	ns	ns	ns	ns
	Control vs SFT	ns	ns	ns	ns	ns	ns	ns	ns	ns	ns	ns	ns	ns
	BFT vs SFT	ns	ns	ns	ns	ns	ns	ns	ns	ns	ns	ns	ns	ns
	Fracture vs control – 180 days post-fracture (n=14)	**0.014**	ns	**0.013**	ns	**0.015**	ns	ns	ns	ns	ns	ns	ns	ns
	Control vs BFT	**0.032**	ns	ns	ns	ns	ns	ns	ns	ns	ns	ns	ns	ns
	Control vs SFT	ns	ns	ns	ns	**0.049**	ns	ns	ns	ns	ns	ns	ns	ns
	BFT vs SFT	**0.004**	ns	**0.004**	ns	**0.005**	ns	ns	ns	ns	ns	ns	ns	ns

Table S3 Complete statistical data of the physicochemical analysis of all samples (perimortem trauma vs postmortem trauma)

	Grouping Variables	Dependent Variable												
		Na	Mg	P	K	Ca	Fe	Zn	Ba	IRSF	API	BPI	BAI	Am/P
Winter	Peri vs post – control samples (n=20)	**0.031**	**0.049**	**0.028**	ns	ns	ns	ns	ns	ns	ns	ns	ns	**0.005**
	Peri vs post – BFT samples (n=24)	ns	ns	ns	**0.001**	ns	ns	**0.008**	ns	ns	ns	ns	Ns	**0.038**
	Peri vs post – SFT samples (n=24)	**0.029**	**0.001**	**0.029**	ns	**0.011**	ns	ns	ns	ns	ns	ns	Ns	**0.034**
Summer	Peri vs post – control samples (n=24)	**0.015**	**0.009**	ns	ns	ns	ns	ns	ns	ns	ns	ns	ns	ns
	Peri vs post – BFT samples (n=24)	ns	ns	ns	ns	ns	ns	ns	ns	ns	ns	ns	ns	ns
	Peri vs post – SFT samples (n=25)	**0.005**	**0.001**	ns	ns	**0.04**	ns	ns	ns	ns	**0.004**	ns	**0.025**	ns

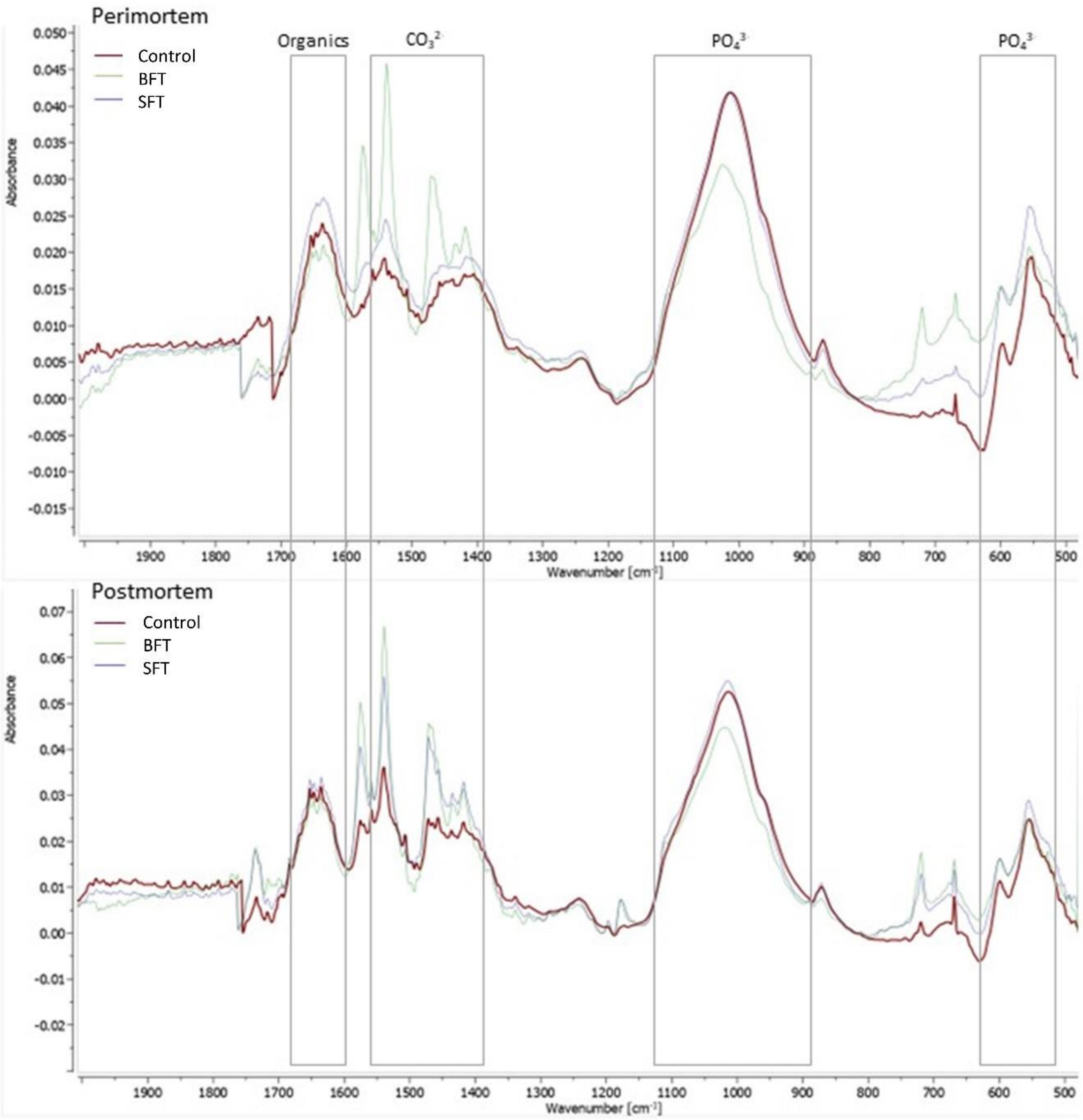

Figure S2 FTIR-ATR sample spectra from the winter study. All spectra were taken at 180 days post-fracture. Peak of interest are highlighted

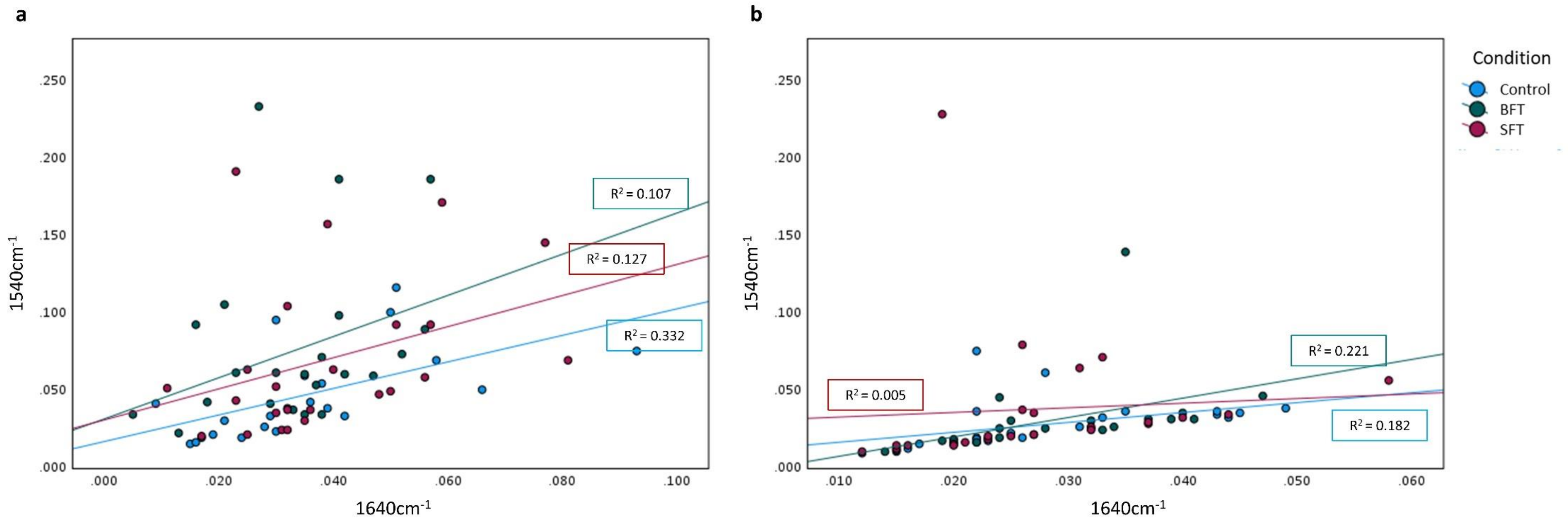

Figure S3 Scatter plots to show the relationship between the absorbance heights at 1540cm^{-1} and 1640cm^{-1} for all samples. **a)** Winter study **b)** Summer study
